# Synthesis of Fe Doped Poly p-Phenylenediamine Composite: Co-Adsorption Application on Toxic Metal Ions (F^−^ and As^3+^) and Microbial Disinfection in Aqueous Solution

**DOI:** 10.3390/toxics9040074

**Published:** 2021-04-01

**Authors:** Elisa Pandelani Munzhelele, Wasiu Babatunde Ayinde, Rabelani Mudzielwana, Wilson Mugera Gitari

**Affiliations:** Environmental Remediation and Nanoscience (EnviReN), Department of Ecology and Resource Management, School of Environmental Sciences, University of Venda, Private Bag X5050, Thohoyandou 0950, South Africa; munzhelele98@gmail.com (E.P.M.); twasiu33@gmail.com (W.B.A.); mudzrabe@gmail.com (R.M.)

**Keywords:** water pollution, arsenic and fluoride remediation, bacterial disinfection, poly para-phenylenediamine composite, adsorption experiments

## Abstract

Water is regarded as an important natural resource to sustain life, and its purification is an important criterion that determines its quality and usefulness. In this study, the incorporation of Fe^3+^ oxide onto a phenylenediamine (pPD) polymer matrix through chemical co-polymerization was prepared, and its arsenite and fluoride removal potentials at optimal conditions from aqueous solution were evaluated. The morphology and structural analysis of the synthesized Fe-doped pPD (Fe-pPD) were comparatively evaluated using the FT-IR, SEM, EDS, and XRD techniques. Fe was successfully incorporated onto pPD matrix as confirmed by different morphological characterizations. The rate of adsorption of F^−^ and As^3+^ onto the Fe-pPD composite best followed the pseudo-second-order kinetic model. The experimental data for both As^3+^ and F^−^ onto the Fe-pPD composite better fit the Freundlich isotherm model at different operating temperatures. Overall, the synthesized composite exhibited a strong affinity towards fluoride uptake (96.6%) than arsenite uptake (71.14%) with a maximum capacity of 6.79 (F^−^) and 1.86 (As^3+^) mg/g. Additionally, the synthesized adsorbent showed some level of antimicrobial activity against common water-borne bacterial. Therefore, the Fe-doped pPD composite has the potential ability for inorganic metal species pollutants remediation and bacterial disinfection in community-level water purification processes.

## 1. Introduction

The most important component of all forms of living organisms on earth is water. Water scarcity has been observed frequently in many parts of the world including Africa [1,2]. Contamination of water resources by multiple pollutants has been known as the most consequential and severe problem worldwide due to natural and anthropogenic activities. The chronic co-existence of pollutants such as fluoride, nitrates, arsenic, and other heavy metal ions, as well as harmful bacteria, by these activities in drinking water sources, has resulted in many complicated life-threatening health effects [3,4]. Arsenic and fluoride have been identified as the most inorganic pollutants in groundwater resources due to water–rock interaction, groundwater recharge, and anthropogenic activities, thus endangering public health [5,6].

Ravenscroft [7] reported that natural arsenic pollution of ground and surface water resources affected several millions of people in at least 70 countries of the world. The most predominant valence state of arsenic is the less toxic arsenates (As^5+^) and the more toxic arsenite (As^3+^), which are found in contaminated water resources [8]. Comparatively, As^3+^ predominantly form in the reducing environment between pH 4 and 10 and exist as neutrally charged, whereas As^5+^ species exist as negatively charged [4]. Chronic contamination of arsenic in drinking water affects various types of biological properties including cardiopulmonary diseases, skin thickening, neurological and gastrointestinal related problems, carcinoma, and arsenicosis found in the human and aquatic ecosystem [9,10].

Furthermore, fluoride (F^−^) is considered the most significant pollutant in groundwater affecting human health adversely across the globe [11]. It has been specified that a little amount of F^−^ is essential for the human body (improves dental health); nevertheless, its excessive intake can cause molting teeth, neurological damages, as well as dental and skeletal fluorosis [12,13]. Fluorosis is an irreversible skeletal disorder with no medical treatment. Comparatively, arsenic exposure in water resources constitutes more danger than fluoride because of its acute toxicity at low concentration [14,15]. Hence, the World Health Organization (WHO) set a threshold for arsenic and fluoride levels in drinking water at 0.01 mg/L and 1.5 mg/L, respectively [10]. Therefore, the detrimental effects associated with the different fates and transport ways of both As^3+^ and F^−^ when ingested have deemed the necessity for the removal of these pollutant ions from contaminated water resources.

Many developing countries are affected by fluoride, arsenite, and microbially polluted water with no affordable purification technologies to enhance drinking water quality. Additionally, various sorbent materials like fly ash, clay, agricultural waste, and polymeric materials have been used for different toxic chemical species removal in waste water [16,17]. The reduction of these pollutants in water has been tested, established, and reviewed by different techniques and materials [18,19,20]. Studies have shown that adsorption technology amongst other techniques has proven to be efficient in remediating these toxic pollutants [21,22].

Recently, researchers have channeled the use of innovative, low-scale, sorbent materials suitable for rural areas in the co-adsorption of As^3+^ and F^−^ from portable water. Metal oxides like iron oxide and iron-embedded sorbents have been used due to their high affinity towards these hazardous inorganic species and pathogen disinfection [23]. However, their applicability has been compromised by the introduction of secondary pollution, where maximum adsorption was at high pH. It is important to note that the removal of these mentioned pollutants must be effective and must not result in other environmental and health implications.

Equally, when selecting a treatment method, it is advisable to choose the best alternative that will have an optimum yield and must be environmentally friendly. The introduction of polymers as an adsorbent in water treatments has been on the rise due to their varied functional groups and structural frameworks. Poly-phenylenediamine of the polyaniline family has been used to assess its adsorption applications for several contaminants such as anionic and heavy metal pollutants [21,24]. Furthermore, the excellent antibacterial property of these metal oxides and polymeric materials to water purification technologies have been reported [25]. The development of metal-metal oxides/polymer to improve and enhance adsorption capacities for efficient water treatment has been an increasing trend lately. Hence, more studies are required to develop and implement less expensive, multifunctional, eco-friendly, sustainable, and advanced technology with high adsorption capacity. In this study, we focus on synthesizing a non-toxic Fe-doped poly-phenylenediamine composite and its potential arsenite and fluoride sorption capacity in groundwater. The adsorption properties of the synthesized sorbent were examined through various experimental conditions, and its adsorption kinetics, isotherms, as well as thermodynamics were also studied and reported.

## 2. Materials and Methods

### 2.1. Chemicals

Poly (p-phenylenediamine), ammonium persulfate ((NH_4_)_2_S_2_O_8_), iron (III) chloride heptahydrate (FeCl_3_ 5H_2_O), sodium fluoride (NaF) and sodium hydroxide (NaOH), NaCl, KCl, HCl, and NaAsO_2_ were of analytical grade and were used without any purifications. The chemicals were obtained from obtained from Sigma-Aldrich (St. Louis, MO, USA) and supplied by Rochelle Chemicals, Johannesburg, South Africa. The prepared solutions of different As^3+^ and F^−^ concentrations were prepared using Ultrapure Milli-Q water S.A.S (Molsheim, France) (18.2 MΩ/cm).

### 2.2. Composite Preparation

#### 2.2.1. Synthesis of Poly p-Phenylenediamine

Poly-pPD was synthesized by a modified method [26,27]. Briefly, 0.015 mol of pPD (1.62 g) was dissolved in 0.1 M HCl (50 mL) and stirred for 3 h on an ice bath. Thereafter, the freshly prepared oxidant solution of ammonium persulfate (HCl (25 mL, 0.1 M, 3.42 g)) was added dropwise into the pPD solution to initiate the polymerization reaction for 30 min. The subsequent solution was mixed continuously under stirring for 24 h at room temperature to allow comprehensive polymerization of the pPD monomer. The pH of the pPD solution was adjusted to 9 by adding 2 M NaOH and shaken at 250 rpm for 30 min. Lastly, 15 mL of acetone was added to stop the polymerization reaction. The obtained solution was further stirred for 10 min to produce the homogenous crude product, which was washed with Ultrapure Milli-Q water and dried under vacuum at 60 °C for 24 h.

#### 2.2.2. Synthesis of Fe-Doped Poly p-Phenylenediamine

A total of 0.015 mol of pPD (1.62 g) was dissolved in 0.1 M HCl (50 mL) and stirred for 3 h on an ice bath. Before the doping process, 0.25 M FeCl_3_ 5H_2_O solution with various percentage weight (2.5, 5, and 10%) was mixed in 20 mL Ultrapure Milli-Q water. Each of these salt solutions was added and mixed separately with the pPD solutions by ultrasonication for 25 min. Next, the freshly prepared ammonium persulfate (3.42 g in HCl (25 mL, 0.1 M)) was added into the solution for 30 min to initiate polymerization of the Fe-pPD composite. The solution was left under stirring at 400 rpm for 24 h to allow the complete formation of Fe-pPD at room temperatures. The pH of the synthesized Fe-pPD was adjusted to 9 with the addition of 2 M NaOH to precipitate the metal hydroxide and shaken at 250 rpm for 30 min. The resulting product was collected by filtration, washed with Ultrapure Milli-Q water, and oven-dried at 60 °C for 24 h.

#### 2.2.3. Optimization of pPD and Fe-pPD

Fluoride (50 mL of 10 mg/L) and arsenite (50 mL of 5 mg/L) solutions were contacted separately with 0.4 g of the modified composite at 250 rpm for 30 min. To assess the pH status of the untreated and treated water, after agitation the resulting pH of each mixture was measured. After the pH measurement, the solution was centrifuged and the supernatants analyzed for residual fluoride using a fluoride ion-selective electrode coupled to an ISE/pH/EC electrode (Thermo Scientific-Orion Versa Star Advanced Electrochemistry meter fluoride ion-selective electrode) (9609 BNWP) (Orion, Waltham, MA, USA). Four standards of fluoride-containing TISAB III with the volume ratio of 1:10 were used to calibrate the fluoride meter, while Metrohm 850 professional ion chromatography (Herisau, Switzerland) was used for the residual arsenite concentration.

Equation (1) was used to calculate the respective percentage removal of As^3+^ and F^−^ in solutions:

(1) $$\%\;\mathrm{metal}\;\mathrm{ion}\;\mathrm{removal}=\frac{Co-Ce}{Co}*100$$

where *Co* and *Ce* are the initial and equilibrium of As^3+^ and F^−^ concentrations, respectively, in mg/L.

Equation (2) was used to calculate the adsorption equilibrium capacity of the adsorbent.

(2) $$q_{e}=\;\frac{Co-Ce}{m}*v$$

where *q_e_*, *m*, and *v* represent the equilibrium capacity of the adsorbent, the mass of the adsorbent in g, and the volume of the As^3+^ and F^−^ in mg/L.

### 2.3. Characterization

The morphological and physicochemical compositions of the synthesized sorbent were assessed using a scanning electron microscope (SEM) (FEI Nova, Brno, Czechoslovakia Republic) with an FEI Nova NanoSEM 230 with a field emission gun equipped with an Oxford Xmax SDD detector operating at an accelerating voltage of 20 KV for the EDS detector (Oxford X-Max with INCA software). The ALPHA Fourier Transform Infra-red spectrum equipped with ATR-Diamond (Bruker, Karlsruhe, Germany) was used to obtain the Infra-red spectrum of the sorbent. Bruker-D8 Powder Diffractometer with a theta-theta goniometer X-ray diffraction (XRD) technique was employed to examine the sorbent structural phase modification. The Thermo Flash 2000 series CHNS/O organic Elemental analyzer (Waltham, MA USA) was used to attain the CHNS results of modified Fe-pPD.

### 2.4. Batch Experiments

The stock solutions of As^3+^ and F^−^ (1000 mg/L) were prepared by dissolving 0.1733 g of NaAsO_3_ and 2.210 g NaF respectively in a 1000 mL volumetric flask using Milli-Q water (18.2 MΩ/cm). The dilution method was used to prepare the working solutions from the stock solution. To examine the effect of contact time and adsorption kinetics, agitation time was varied from 0.5 to 120 min. A Fe-pPD composite dosage of 0.4 g/50 mL and an initial concentration of 5 and 10 mg/L (As^3+^ and F^−^), respectively, was maintained. After agitation, the resulting mixtures were centrifuged at 250 rpm for 20 min. To evaluate the effect of the adsorbent dose, the sorbent dosage was varied from 0.1 to 0.4 g/50 mL. To determine the effect of initial concentration, adsorption isotherms, and thermodynamic process of adsorbate (F^−^ and As^3+^) adsorption, the respective adsorbate concentration was varied from 5 to 100 mg/L at temperatures of 298, 323, and 343 K.

The effects of pH were assessed by adjusting the initial solution pH (2–12) using 0.01 M NaOH and 0.01 M HCl. Additionally, the effects of co-existing ions (F, Cl, NO_3_^−^, CO_3_^2−^, SO_4_^2−^) on As^3+^ and F^−^ were evaluated at room temperature. All experiments were conducted in triplicate, and the mean values were reported. The pHpzc of the synthesized adsorbent was estimated using the solid addition method as described by Gitari et al. (2017) [28]. Equations (1) and (2) were used to determine the percentage adsorbate removal and adsorption capacity, respectively.

### 2.5. Adsorption Kinetics

The As^3+^ and F^−^ adsorption kinetics were studied at initial concentrations of 5 and 10 mg/L, respectively. The experimental data were analyzed using the non-linear equation of pseudo-first-order and pseudo-second-order models as well as intraparticle diffusion (Equations (3)–(5)) [29,30,31]:

(3) $$qt=qe\left(1-e^{-kit}\right)$$

(4) $$qt=\frac{qe^{2}\;k_{2}t}{1+k_{2}\;q_{e}t}\;$$

(5) $$qt=k_{t}t^{0.5}+ci$$

where *qe* (mg/g) and *qt* (mg/g) are the mass of the adsorbate ion units at equilibrium and at time *t* (min), respectively. *K*_1_ (min^−1^) and *K*_2_ (g·mg^−1^ min) are the respective rate constant values of the pseudo first- and second-order. *Ki* (mg/g min^−1^) represents the rate constant of the intraparticle diffusion model obtained from the slope of *t*^0.5^ vs. *qt* and *Ci* (the constant attained from the intercept which reflects the thickness of the boundary layer). The higher the intercept, the greater the boundary layer effect [32].

The Elovich linear equation (Equation (6)) has general application to chemisorption kinetics.

(6) $$qt=\beta \ln\left(\partial \beta \right)+\beta \ln t$$

The equation was used to validate that chemisorption is the limiting step for fluoride and arsenic uptake. The Elovich model is usually used to validate the nature and type of sorption process(es) occurring at the adsorbing composite surface. *qt* is the amount of the pollutant ion adsorbed at time *t* (mg/g), *α* is the constant relative to the rate of chemisorption, and *β* is the rate constant which shows the extent of surface coverage. These two constants (*α* and *β*) are obtained from the intercept and slope of the plot from Equation (6).

### 2.6. Adsorption Isotherms

The adsorption isotherms were calculated using the theoretical Langmuir and empirical Freundlich isotherms [33,34]. The Langmuir isotherm model assumes monolayer interaction between the adsorbate molecules bound to the adsorbent surface during adsorption. The non-linearized data are shown in Equation (7).

(7) $$qe=\frac{q_{m}K_{L}C_{e}}{1+K_{L}C_{e}}$$

*C_e_*, *Q_e_*, *Q_m_* and *K_L_* represent the equilibrium concentration (mg/L), adsorption capacity (mg/g), theoretical maximum adsorption capacity (mg/g), and the Langmuir constant related to enthalpy of adsorption (L/mg), respectively. Equation (8) was used to express the dimensionless constant separation factor of the Langmuir isotherm model *R_L_* (when *R_L_* = 1 irreversible, 0 < *R_L_* < 1 favorable, *R_L_* = 1 linear, and *R_L_* > 1 unfavorable).

(8) $$R_{L}=\frac{1}{1+k_{L}C_{i}}$$

The Freundlich isotherm model suggests a mutual interface among the chemical species being adsorbed onto the multilayered surface of the adsorbent. The non-linear equation of Freundlich is expressed as Equation (9):

(9) $$qe=K_{f}Ce^{\frac{1}{n}}$$

The Freundlich constant associated with adsorption capacity and the adsorption intensity is represented by the *K_f_* values and 1/*n*, respectively. When 0 < 1/*n* < 1, the adsorption is favorable; when 1/*n* = 1, the adsorption is irreversible; and when 1/*n* > 1, the adsorption is unfavorable.

The Dubinin Radushkevich (D-R) model (Equation (10)) was employed using the experimental data. D-R model assumption gives details about the porous nature of the adsorbent as well as adsorption energy. The obtained value of adsorption energy provides information as to whether the adsorption process is physical or chemical [35].

(10) $$Inqe=Inqo-\beta e^{2}$$

where *qe* and *qo* represent the number of ions adsorbed per unit mass of adsorbent (mg/g), the maximum adsorption capacity; *β* represents the activity coefficient useful in obtaining ε (Polanyi potential), and mean sorption energy *E* (kJ/mol) in Equations (11) and (12) respectively.

(11) $$\varepsilon =RT\left(1+\frac{1}{Ce}\right)$$

(12) $$E=\sqrt{\frac{1}{2\beta }}$$

*R* denotes the gas constant (J/mol K), and *T* is the temperature (*K*). *qo* and *β* (mol^2^/kJ^2^) can be calculated respectively from the intercept and the slope of the plot of *Inqe* vs. ε^2^.

### 2.7. Goodness-of-Fit Valuation

The model goodness-of-fit calculations was done to validate the fitness of the kinetics, and isotherm models were obtained from the experimental data through the coefficient of determination (*R*^2^) (Equation (13)), root mean square error (*RMSE*) (Equation (14)), and the sum of the squared errors (*SSE*) (Equation (15)).

(13) $$R^{2}=1-\frac{\sum \left(q_{e,\;exp}-q_{e,\;calc}\right){}^{2}}{\sum \left(q_{e,\;exp}-q_{e,\;mean}\right){}^{2}}$$

(14) $$RMSE=\sqrt{\frac{1}{n-1}\overset{n}{\underset{i=1}{\sum }}\left(q_{e,\;exp}-q_{e,\;calc}\right){}_{i}^{2}}$$

(15) $$SSE=\overset{n}{\underset{i=1}{\sum }}{\left(q_{e,calc}-q_{e,\;exp}\right)}^{2}$$

where *q*_*e,calc*_ is the theoretical concentration of adsorbate on the adsorbent, which has been calculated from one of the isotherm models. *qe* and *i* are the experimentally measured adsorbed solid-phase concentration and the number of experiments respectively.

### 2.8. Antimicrobial Activity Test

The antimicrobial potency of the synthesized Fe-pPD and pPD was evaluated using the standard Agar-Well disc diffusion method by observing the observed zone of inhibition (mm). Medium 1 agar plates were divided into half; 1–5 mL pipette tips were used to punch a small circle to add the adsorbent. A volume of 50 µL of the bacterial strains (*E. coli*, ATCC 25,922 IN; *S. aureus*, ATCC 259,231 Tm; and *K. pneumoniae*, ATCC 700603) was inoculated into the sterile medium 1 agar. Then, 50 µL of 1 mL/0.01 g of the sorbent was deposited into the punched circles and incubated for 24 h at 37 °C. The minimal zone of inhibition was observed and measured.

## 3. Results and Discussion

### 3.1. Adsorbent Optimization

**Figure 1:**
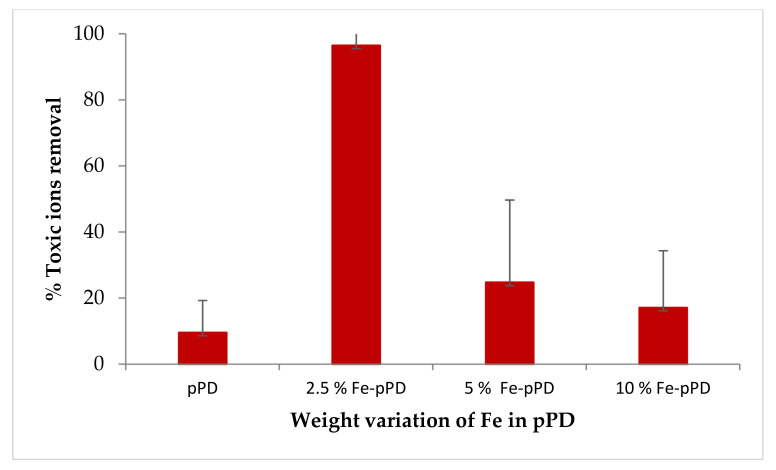
Percent removal of pPD and % Fe in pPD adsorbents for As^3+^ and F^−^ uptake (initial concentration: 10 mg/L, adsorbent dose 0.4 g, and contact time 30 min at 297 K).

Figure 1 shows the adsorption capacities of the chemical species by varying the weight percentages of the synthesized materials with initial concentrations of 10 mg/L (F^−^) and 5 mg/L (As^3+^), a material dosage of 0.4 g at room temperature with a contact time 30 min at a shaking speed of 250 rpm. The respective sorption experiment was carried out by observing the effects of weight variation (*v*/*v*%) of Fe in the pPD matrix (2.5, 5, and 10%) compared with the bare pPD.

Figure 1 portrayed the bare pPD polymer having the lowest pollutant removal potential, where only 10% of the initial concentration was removed. However, when pPD was doped with Fe, the sorption capacity of the composite improved considerably. A rise in the adsorption capacity might be due to the reason that iron-based sorbents have high binding infinity towards inorganic pollutants (As and F) [36]. Additionally, the introduction of new functional groups (accepting electronic systems in the pPD matrix) enhances the adsorption efficiency when combined with the Fe ions on the modified composite (Fe-pPD). The modification of the pPD through the introduction of FeCl_3_ has influenced the formation of an emeraldine hydrochloride Fe doped poly-(para-phenylenediamine) (R-FeCl_3_) [37]. Thus, the expected mechanism for the toxic metal ions removal might be through ligand exchange [38], where the metal ions are attached to Fe replacing chloride ions. Thus, the introduction of Fe metal oxide into the pPD network significantly improved the surface area and ionic state by increasing the active binding sites and solubility, which had improved the adsorption efficiency of the Fe-pPD. Consequently, an improvement in surface area was further validated by the BET analysis, confirming the high availability of the active binding site. From the data, 2.5% was chosen as better adsorbent and further used for arsenic and fluoride removal. The interaction of the Fe metal oxide-modified polymeric composite was a consistent determinant in the enhancement of the metal ions uptake.

### 3.2. Characterization of the Sorbent

#### 3.2.1. FIR Results

**Figure 2:**
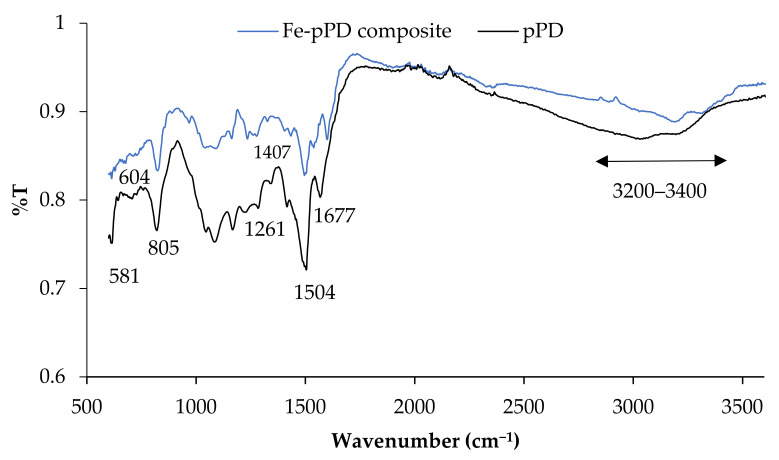
FTIR spectra of bare pPD and Fe-pPd composite.

The FTIR spectra of the Fe-pPD composite were analyzed to determine the functional groups and any transition within the composite spectrum due to the incorporation of Fe metal oxide. Figure 2 shows the FTIR spectra of pPD and Fe-pPD composite with their respective peaks. The pPD spectrum shows a typical broad band between 3200 and 3400 cm^−1^ attributed to the presence of stretching vibration of N-H group and hydroxyl group (-OH) stretching from physically adsorbed H-O-H bonded to the surface [26,39]. The appearances of strong peaks at 1677 and 1504 cm^−1^ relate to the C=C and C=N stretching vibrations of the phenazine ring [40]. The C-N-C stretching vibrations of benzenoid and quinoid imine units are recognized by the peaks at 1407 and 1261 cm^−1^ with the characteristic of C-H out-of-plane bending vibrations of benzene bases in the phenazine skeletons, which are the bands at 805 and 581 cm^−1^ respectively [41]. Weak vibrations at ~604 cm^−1^ are typical of Fe-O bonding across the polymer composite.

#### 3.2.2. SEM-EDS Results

**Figure 3:**
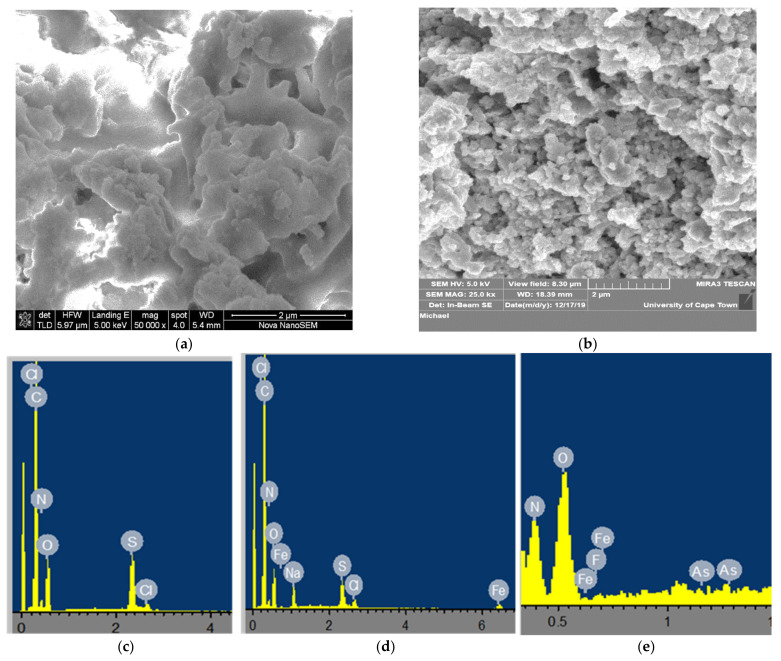
SEM-EDS results: (**a**,**b**) SEM images of bare pPD and Fe-pPD; (**c**) EDS spectra of bare pPD; (**d**) Fe-pPD EDS spectra; (**e**) EDS spectra of the co-adsorbed As and F ions on the Fe-pPD composite.

Generally, the surface morphology of polymer-based metal-oxide composites is dependent on the type of polymers of the phenylenediamines as well as the oxidant used during the synthesis [42]. Figure 3 displays the SEM-EDS micrographs of bare pPD and the Fe-pPD composite. The morphology of the bare pPD (Figure 3a) was found to be a globular arrangement, while structurally the Fe-pPD (Figure 3b) depicts a well-arranged aggregation due to interaction and anchoring of Fe surfactant across the polymer matrix of pPD. Both materials were measured possessing average particle size ranges between 2 and 20 µm. The successful incorporation of the metal oxide within the polymer backbone in Fe-pPD as compared to the bare pPD polymer framework was confirmed by the various elemental compositions present in the EDS mapping analysis (Figure 3c,d). The presence of Fe in the Fe-pPD composite together with other elements exhibited in pPD as shown in the EDS spectra supported the formation of the Fe-pPD composite. Additionally, Figure 3e portrayed the potential ability of the Fe-pPD composite in the simultaneous removal of As^3+^ and F^−^, which was validated by the presence of As and F in the EDS spectrum.

#### 3.2.3. XRD Results

**Figure 4:**
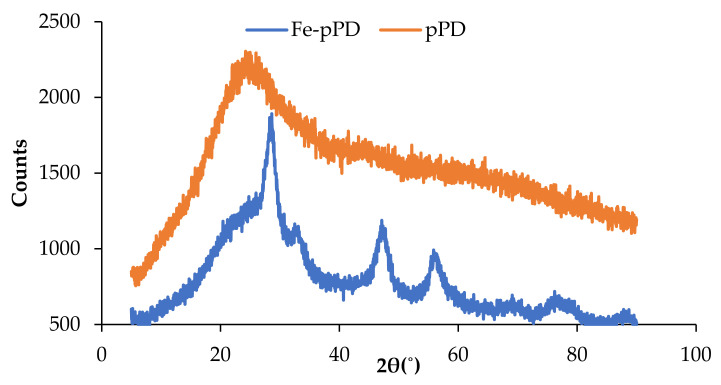
XRD diffractogram of pPD and Fe-pPD composite.

The structural phases of the bare pPD and Fe-doped pPD composite are shown in Figure 4. The X-ray diffractogram of bare pPD shows a large, broad diffraction peak around 25°, signifying the amorphous nature of pPD synthesized by the oxidative polymerization process. A similar morphological phase of the bare pPD was reported elsewhere [43]. The transformation from the amorphous state exhibited by the bare pPD network to the crystalline form of Fe-doped pPD composite was affirmed by the emergence of four distinct new peaks (2θ = 28.9°, 33.65°, 48.5°, and 57.9°). The diffraction peak patterns of Fe oxides were indexed to 33.65° and 58.65°, which were the typical peaks associated with two-line magnetite (ICDD database).

#### 3.2.4. BET Results

**Figure 5:**
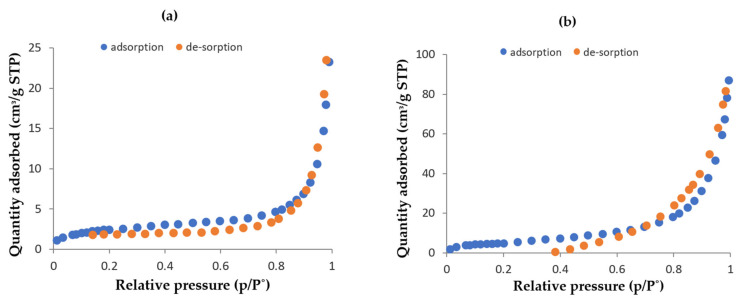
BET adsorption and desorption plots of (**a**) bare pPD and (**b**) Fe-pPD.

The BET analysis of the bare pPD and Fe-pPD was done to evaluate the change in surface area and to determine the porous nature of the material. The results from the BJH pore size distribution portrayed an improvement in surface area, pore diameter, and volume from the pPD to the Fe-pPD composite (Table 1). Figure 5a,b portrayed the adsorption–desorption profile of the bare pPD and Fe-pPD composite, respectively. The increase in the surface area might be associated with the decrease in particle sizes pPD (60.99 nm) > Fe-pPD (32.62 nm), thus aiding in an increase in adsorption capacity. Additionally, the resulting pore diameters affirm the mesoporosity of the composite.

#### 3.2.5. CHNS Analysis Results

Table 2 shows the attained CHNS elemental composition in the Fe-pPD composite before and after adsorption application. As shown, the percentage of CHN increased after the metal ions sorption, whereas S content was below the detection limit. The increase in CHN content after application might be because the polymers polyaniline results in chain growth due to condensation polymerization, thus increasing the percentage of CHN [44]. However, the S content in the composite before the application may be due to the use of ammonium persulphate as an oxidant during the polymerization processes. It is known that ammonium persulphate tends to easily dissolve in water, thus indicating the reduction in S percentage after application.

### 3.3. Adsorption of Fe-pPD Composite

#### 3.3.1. Effect of Contact Time on As^3+^ and F^−^ Sorption Using Fe-pPD

**Figure 6:**
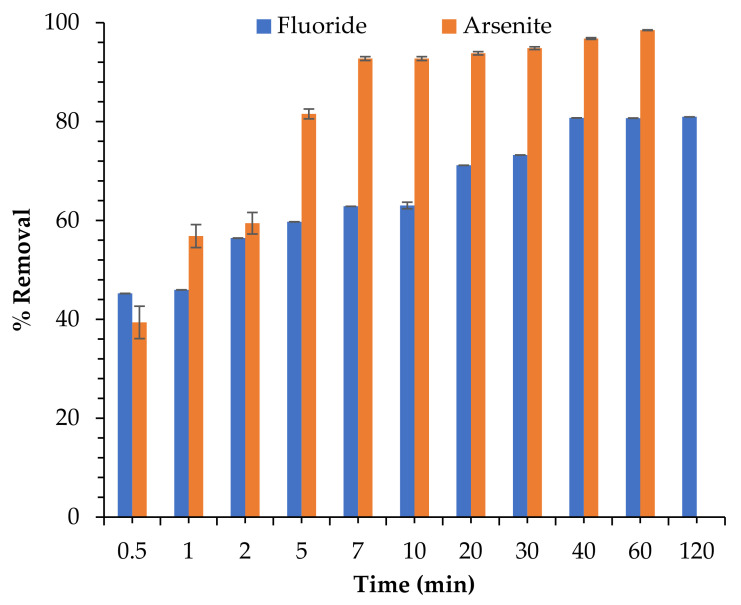
The significance of contact time on F^−^ and As^3+^ sorption using Fe-pPD. (Initial F^−^ and As^3+^ concentration: 5 and 10 mg/L, respectively; adsorbent dose: 0.4 g; solution volume: 50 mL; shaking speed: 250 rpm at 297 K).

Figure 6 displays the experimental data of contact time on the simultaneous uptake of As^3+^ and F^−^ in aqueous solution by the synthesized Fe-pPD composite studied between 0.5 and 120 min. In both sorption processes of As^3+^ and F^−^, the percentage removal increased exponentially with contact time in the first 40 min and subsequently slowed down to a flattened peak to attain sorption equilibrium. The maximum percentage removal for As^3+^ and F^−^ was recorded at 99% (60 min) and 81% (120 min) respectively. The initial increase in As^3+^ and F^−^ sorption might be attributed to the availability of active binding sites of the Fe-pPD surface to take up these ions in solution, which at equilibrium time were unavailable at saturation period, hence the low uptake efficiency by the adsorbent at the latter stage of the process. The equilibrium contact time for As^3+^ and F^−^ sorption was 40 min. The optimal time was further used for subsequent batch experiments.

#### 3.3.2. Adsorption Kinetics

**Figure 7:**
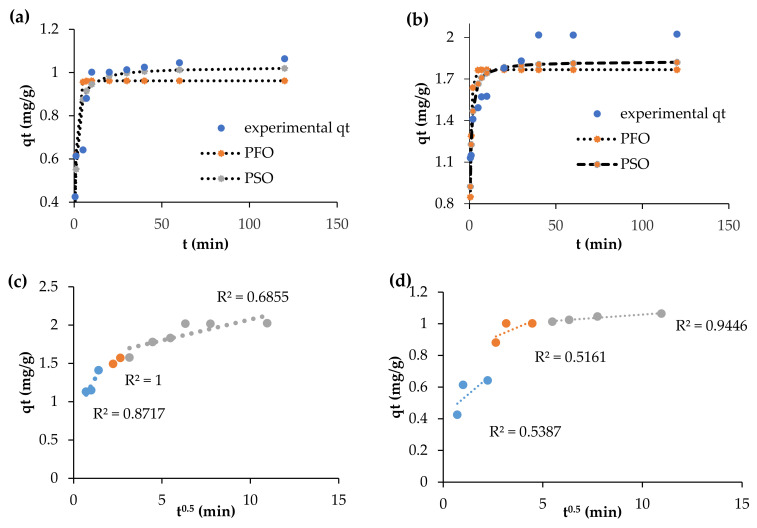
Kinetic models (**a**,**b**): pseudo-first- and second-order; (**c**,**d**): intra-particle diffusion of F^−^ and As^3+^ respectively onto the Fe-pPD composite.

The estimated parameters for the reaction (pseudo-first- and second-order) and diffusion-based (intra-particle) kinetic models data for both F^−^ and As^3+^ are presented in Table 3. The kinetic plots (Figure 7a,b and data for F^−^ and As^3+^ adsorption onto the Fe-pPD composite best followed the pseudo-second-order as validated by the *R*^2^ for As^3+^ (1) > *R*^2^ for F^−^ (0.923). Thus, chemisorption is the limiting step for fluoride and arsenite removal. From a statistical point of view, the *χ*^2^ and RMSE pseudo-first-order model presented low values for both F^−^ and As^3+^.

Thus, this implies favorability and suggesting the uptake mechanisms of both pollutants by the Fe-pPD composite is associated with the chemisorption process. The obtained coefficient of determination (*R*^2^) of the linearized Elovich (Table 4) was greater than that of both the pseudo-first- and pseudo-second-order (Table 3). Thus, this implies favorability of chemisorption of the solid–liquid adsorption process.

Generally, the mass transfer process of the solid–liquid phase sorption is normally represented by the external mass transfer, intraparticle diffusion, as well as adsorption on the active binding sites consecutively. Figure 7c,d shows the intra-particle diffusion simulated plots of both As^3+^ and F^−^ uptake by the Fe-pPD composite. For an adsorption process to be controlled by this model, the intra-particle diffusion plot must give a straight line passing through the origin.

However, in this work, the intra-particle plot attained from the adsorption data (Figure 7c,d) shows that the adsorption process occurred due to multiple steps. This was consistent with the results for both the As^3+^ and F^−^ adsorption processes, indicating the uptakes of both pollutants were not controlled by only intra-particle diffusion. As shown on the plot, three defined phases, which deviate from the origin, occurred for both As^3+^ and F^−^ uptake by the Fe-pPD composite. These phases show the systematic mechanisms of both the As^3+^ and F^−^ species in solution occurring due to the boundary layer diffusion, intraparticle pore diffusion, and on the active sites across the external Fe-pPD composite surface. Comparatively, the rates for phase 1, 2, and 3 propose that the sorption process occurred quickly on the layer and was controlled by intraparticle diffusion for F^−^, whereas on As^3+^ it was the attachment of As^3+^ on the internal surface of the adsorbent due to the difference in the rate of mass transfer in the initial and final phase of adsorption based on the respective coefficient of determination values (Table 4). The positive value of Ci indicates that intra-particle is the main mechanism for adsorption, and external diffusion occurred to some extent.

#### 3.3.3. Effect of Fe-pPD Composite Dose on As^3+^ and F^−^ Sorption

**Figure 8:**
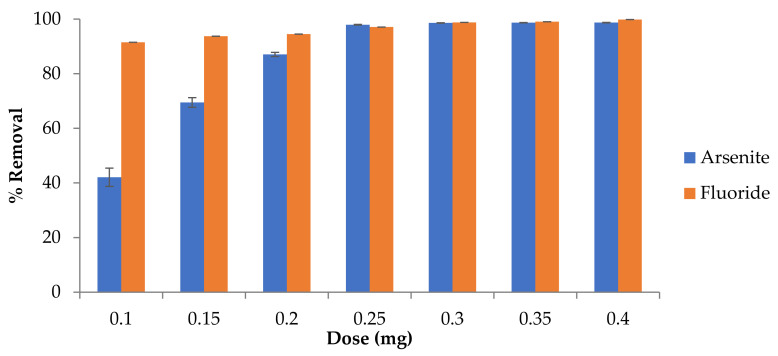
Effect of adsorbent dose on As^3+^ and F^−^ sorption. Initial F^−^ and As^3+^ concentrations: 5 and 10 mg/L; adsorbent solution volume: 50 mL; contact time: 40 min; shaking speed: 250 rpm at 297 K.

The response of the Fe-pPD composite (adsorbent) dose on As^3+^ and F^−^ ions removal is presented in Figure 8. The adsorbent dose was varied from 0.05 to 0.4 g. The attained data portrayed an increase in As^3+^ and F^−^ removal with increasing doses of the Fe-pPD composite. The gradual increase in removal ability with a rapid increase in adsorbent load might be attributed to an increase in binding active sites on the adsorbent surface available for As^3+^ and F^−^ adsorption. Thus, Fe-pPD can effectively remove As^3+^ and F^−^ ions as the dose increases. Equally, the lower As^3+^ and F^−^ sorption by Fe-pPD at low adsorbent is due to saturation of the available active sites on the adsorbent surface of the adsorbent. Thus, this implies the significance of the adsorbent dose towards As^3+^ and F^−^ uptake. Therefore, the maximum removal uptake was recorded, and the optimum dosage was 0.25 g and 0.2 g/50 mL for As^3+^ and F^−^, respectively. These optimal doses were used for subsequent batch adsorption experiments.

#### 3.3.4. Effect of Initial Concentration

**Figure 9:**
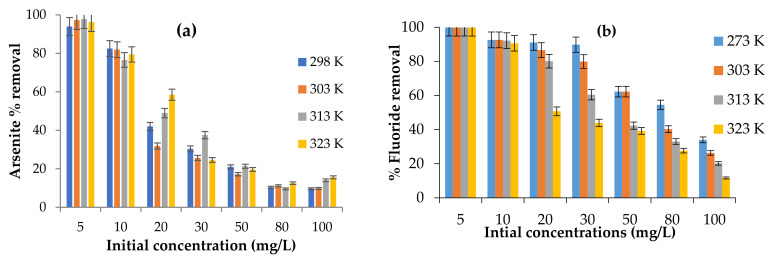
Effect of initial concentrations on (**a**) As^3+^ and (**b**) F^−^ sorption at different working temperatures. Initial F^−^ and As^3+^ concentration: 5 and 10 mg/L; adsorbent dose: 0.25 g and 0.2 g; solution volume: 50 mL; contact time: 24 h; shaking speed: 250 rpm; and temperature ranges: 298–323 K.

The significance of the initial adsorbate concentration on adsorption efficiency of the Fe-pPD composite was evaluated between 5 and 100 mg/L at different operating temperatures (Figure 9a,b). As shown from the obtained data, the percentage removal of both As^3+^ and F^−^ by the Fe-pPD composite decreased with a rise in the initial concentration. The reduction in the removal efficiency by both As^3+^ and F^−^ species with increasing initial concentration may be due to the increasing number of these species in the solution. Thus, saturation of the binding sites on the Fe-pPD adsorbent surface area was indicated. This trend was consistent throughout the different operating temperatures and can be ascribed to a decrease in the mass transfer movement, which allows better binding interaction between both ions (As^3+^ and F^−^) and the functional binding sites of the Fe-pPD composite.

#### 3.3.5. Adsorption Isotherm Models

**Figure 10:**
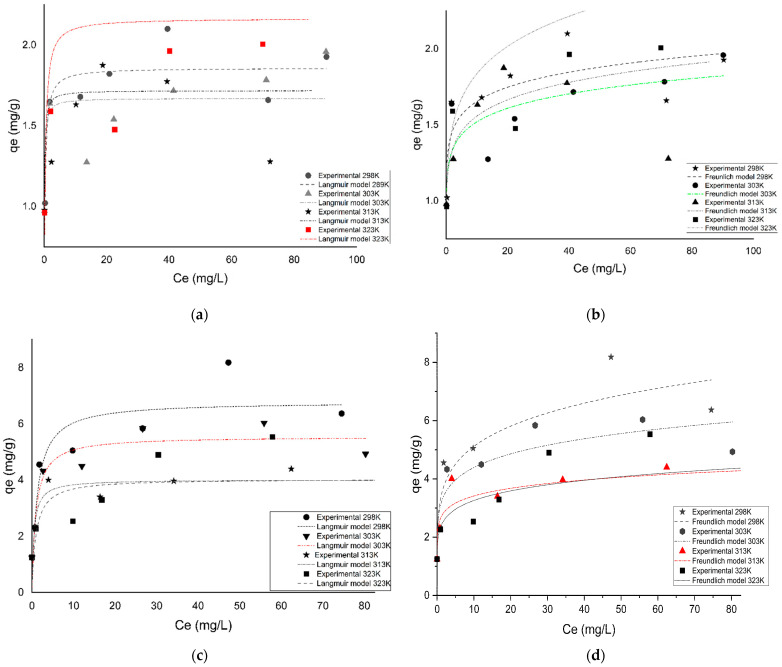
(**a**,**b**) As^3+^ for Langmuir and Freundlich isotherm model plots; (**c**,**d**) F^−^ for Langmuir and Freundlich isotherm model plots.

The non-linearized plots with the respective parameters of both the Langmuir and Freundlich models for As^3+^ and F^−^ adsorbed sorption onto Fe-pPD at different temperatures are summarized in Figure 10a,b and Table 5. Evidently from the coefficient of determination values (*R*^2^), adjusted correlation coefficient values (adjusted *R*^2^), R chi-squared values (*χ*^2^), and residual sum of squares (RSS) (Table 5), the adsorption process of both As^3+^ and F^−^ by Fe-pPD followed the Freundlich isotherm model with higher affinity for F^−^ than As^3+^ based on the maximum adsorption capacity (Q_m_). Thus, this indicates the heterogeneity adsorption phenomenon at the sorbate–sorbent interphase. The maximum adsorption capacities increased for As^3+^ and decreased for F^−^ uptake as the temperature increased. Thus, this suggests that adsorption infinity is high at high temperature for As^3+^, whereas for F^−^ it is favorable at low operating temperature. Moreover, the n values within the range of 1 and 10 validate the favorability of the sorption processes. 

Additionally, the D-R model was also plotted to determine the effect of the porous nature of the composite as well as the mean free energy of the adsorption process(es). Table 6 displays the various model parameters of Fe-pPD composite sorption of As^3+^ and F^−^. A reduction in the Q_max_ value of F^−^ with increasing temperature was observed, showing that adsorption infinity is high at low temperatures. Whereas, As^3+^ data revealed that adsorption infinity was high at high temperatures, as shown in Table 6. The obtained Polanyi potential (E) value was < 8 kg/mol; thus, the adsorption process occurred through a physical process with the surface and composition of the composite.

#### 3.3.6. Thermodynamics

Thermodynamic parameters such as enthalpy changes (Δ*H*° (kJ/mol^−1^)), entropy changes (Δ*S*° (kJ/mol^−1^)), and Gibbs free energy changes (Δ*G*° (kJ/mol^−1^)) were used to determine the spontaneity, type of reaction, and the degree of randomness during the uptakes of both As^3+^ and F^−^ by the Fe-pPD. These parameters were obtained from the plot of 1/T vs. ln *K_C_* and calculated using the following equations:

(16) $$\ln K=\frac{\Delta H^{*}}{R}+\frac{\Delta S^{{}^{\circ}}}{R}$$

(17) $$\Delta G=\Delta H^{{}^{\circ}}-T\Delta S^{{}^{\circ}}$$

*K*, *R*, and *T* represent the equilibrium constant, gas constant (8.134 kJ/mol^−1^ K^−1^), and solution temperature (*K*), respectively.

Table 7 shows the obtained relative thermodynamic values for the metal ion adsorption process. From the tabulated parameters, ∆G° values were calculated to be negative for F^−^ and As^3+^ sorption processes. Thus, this indicates the feasibility and spontaneity for both As^3+^ and F^−^ uptake by the Fe-pPD. In both removal processes, the ∆*G*° values decreased with an increasing temperature, which indicates the favorability of the sorbate-sorbent mechanisms. Thermodynamically, the removal process for both As^3+^ and F^−^ by the Fe-pPD was endothermic with an increase in the degree of randomness, as validated by the positive values of ∆*H*° and ∆*S*° respectively.

#### 3.3.7. Effect of pH on As^3+^ and F^−^ Sorption and Point of Zero Charge

**Figure 11:**
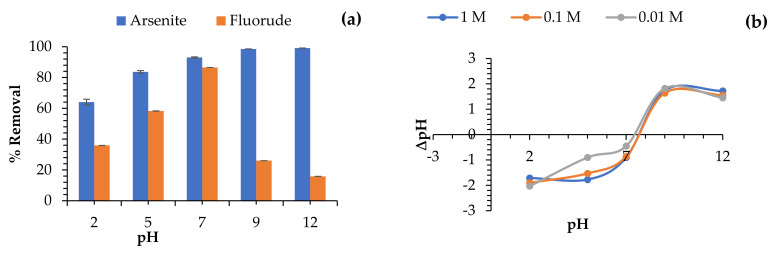
Effect of pH (**a**) and pH_pzc_ (**b**). Initial As^3+^ and F^−^ concentration: 5 and 10 mg/L; adsorbent dose: 0.25 and 0.2 g; solution volume: 50 mL; contact time: 40 min; shaking speed: 250 rpm; and temperature: 297 K.

The effects of pH and ionization abilities of the composite on As^3+^ and F^−^ species sorption were examined by changing the reaction solution pH (between 2 and 12), and the obtained results are shown in Figure 11a,b. As^3+^ and F^−^ uptake in aqueous solution is widely reported to be highly pH-dependent. The point of zero charge of Fe-pPD was examined, and the attained result is shown in Figure 11b. The pH at the point of zero charge is defined as the pH at which the net charge of the adsorbent surface is equal to zero. However, the importance of this phenomenon is that the surface will be positively charged at solution pH values less than pH_pzc_, whereas negatively charged when the pH of the solution is greater than the pH_pzc_. From the obtained results, the pH_pzc_ was 7.

As revealed on the plot (Figure 11a), it was observed that As^3+^ uptake by the Fe-pPd composite increased with increasing the solution pH. From the obtained results, the optimal As^3+^ removal pH was reported to be 9. Relatively, an increase in As^3+^ removal is due to electrostatic attraction, which is attributed to an increase in OH^−^ ions in the sorbent-sorbate solution and ion exchange between the adsorbent surface charges and adsorbates charges at pH above the pH_pzc_. The low As^3+^ uptake at the acidic region is due to the force of repulsion between the sorbent surface and pollutant ions charges as well as the competition between the hydrogen ions and As^3+^ ions. Recently, it has been reported that As^3+^ is an oxy ion that speciates at different pH under reducing and oxidizing conditions. Due to the speciation of arsenite in solution, at pH > 9 As^3+^ occurs as neutrally charged and negatively charged at pH < 9 [45]. Hence, there is less repulsion force aiding to a steady increase in its removal by the composite.

Additionally, it was detected that F^−^ removal by the Fe-pPD increased in less acidic to neutral pH and decreased as the pH increased to alkalinity. An optimal F^−^ removal pH was reported to be 7, where about 85.51% removal was observed. An initial increase in F^−^ removal is due to ion exchange and electrostatic attraction between the adsorbate and adsorbent surface charges, as validated by the pH_pzc_. However, as detected from the pH_pzc_ phenomenon (~pH 7), reduction in F^−^ uptake is attributed to repulsion force between the adsorbent surface charge and the F^−^ ions and competition between OH^−^ ions and F^−^ ions.

#### 3.3.8. Effect of Co-Existing Ions

**Figure 12:**
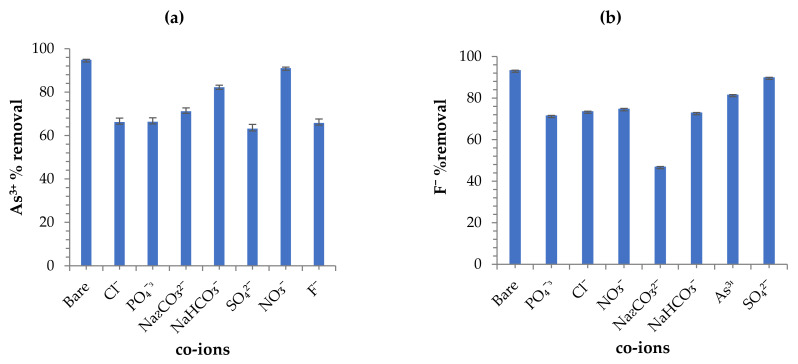
(**a**,**b**) Effect of co-existing ions plots for As^3+^ and F^−^ (initial F^−^ and As^3+^ concentration: 5 and 10 mg/L respectively; adsorbent dose: 0.25 and 0.2 g; solution volume: 50 mL; contact time: 24 h; shaking speed: 250 rpm; and temperature: 297 K).

The occurrences of co-existing ions such as phosphates, sulphate, chlorine, fluoride, arsenite, carbonates nitrates, etc., in water might pose greater or minimum effect upon As^3+^ and F^−^ ions uptake by the composite in aqueous solution. Figure 12 clearly shows that as the concentration of co-existing ions increased, adsorption capacity is reduced. Hence, reduction in pollutants ion removal is due to the capacity of the competing ion with As^3+^ and F^−^ ions for the present binding active adsorbent surface sites [46]. Additionally, the co-occurrence of existing ions reduced interaction mobility of the adsorbate ions with active sites, thus causing high coulombic repulsion forces. As attained from Figure 12a, the results revealed that SO_4_^2−^, F^−^, PO_4_^−^, and Cl^−^ posed a great effect on As^3+^ removal, whereas CO_3_^2−^ and NO_3_^−^ posed minimal effect. Additionally, the obtained F^−^ results revealed greater CO_3_^2−^ effect, whereas SO_4_^2−^, As^3+^, PO_4_^−^, and Cl^−^ were minimal as shown in Figure 12b. Equally, the effect order can be summarized in the following way: arsenite = SO_4_^2−^ > F^−^ > PO_4_^−^ > Cl^−^ > CO_3_^2−^ > HCO_2_^−^ > NO_3_^−^ and fluoride = CO_3_^2−^ > PO_4_^−^ > HCO_2_^−^ > Cl^−^ > NO_3_^−^ > As^3+^ > SO_4_^2−^.

#### 3.3.9. Regeneration

**Figure 13:**
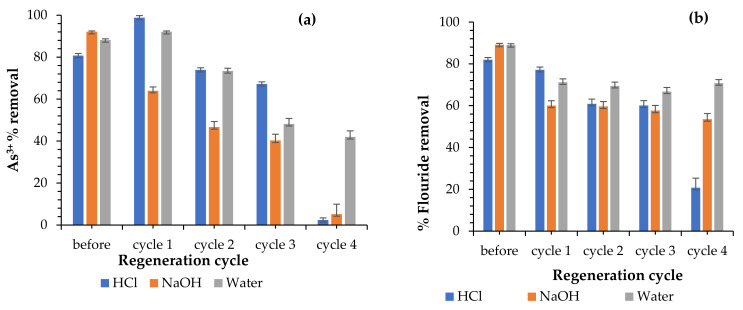
Regeneration results (**a**) As^3+^ and (**b**) F^−^ (initial F^−^ and As^3+^ concentration: 5 and 10 mg/L; adsorbent dose: 0.25 and 0.2 g; solution volume: 50 mL; contact time: 40 min; shaking speed: 250 rpm; and temperature: 297 K).

The reusability and economic viability of the Fe-pPD adsorbent were evaluated through the regeneration experiment, as shown in Figure 13. The efficiencies of the adsorbent in the uptake of As^3+^ and F^−^ ions in an aqueous solution were studied for up to four regeneration cycles. As^3+^ and F^−^ percent removal as a function of the regeneration cycle using 0.01 M NaOH and 0.01 M HCl as well deionized water was used as regenerants. From the obtained results in Figure 13a,b, the As^3+^ and F^−^ sorption potential of the regenerated material reduced with increasing regeneration cycles when using HCl, NaOH, and H_2_O regenerants. The reduction in As^3+^ and F^−^ uptake might be due to a reduction in adsorbent dose probably because the polymeric composite was irreversibly and oxidized [27] as well as the dissolution of Fe oxide in the regenerants. However, based on the attained results, the study showed that the adsorbent can be an economically viable and efficient adsorbent to eradicate water pollution.

### 3.4. Removal Mechanism

Based on the experimental results, different mechanisms are involved in fluoride and arsenite removal by the Fe-pPD composite. However, the kinetics models, isotherm models, thermodynamics, effect of pH, as well as point of zero charges were examined to determine the pollutant removal mechanisms. The kinetics data revealed that the removal process includes physio-sorption, chemo-sorption, and internal attachment of solid/liquid interface. This was further validated by the D-R isotherm model, thermodynamics, as well as the BET analysis results. Furthermore, it was reported that electrostatic attraction and ionic exchange are the removal mechanisms, as validated by the results of the effects of pH and pH_pzc_.

### 3.5. Antimicrobial Potency

**Figure 14:**
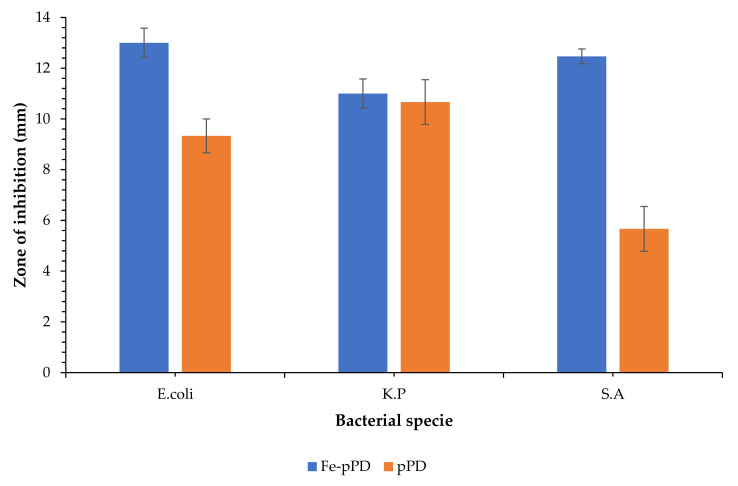
Antimicrobial activity of the Fe-pPD composite.

The antimicrobial activities of pPD and Fe-pPD composites against *Escherichia coli* (*E. coli*), *Klebsiella pneumonia* (K.P), and *Staphylococcus aureus* (S.A) are displayed in Figure 14. Comparatively, bare pPD inhibited about 9, 10, and 6 mm, whereas the modified Fe-pPD composite inhibited about, 13, 11, and 12.5 mm. Thus, the modified composite has more susceptibility towards the concerned pathogens compared to bare pPD. The antimicrobial property of the synthesized adsorbent might be due to the existence of co-coordinatively unsaturated Fe metal of the composite material, which generates reactive oxidative species to assist cellular distraction and cell death [47]. Furthermore, different functional groups, especially the amines, within the Fe-pPD composite are also responsible for the denaturation of cell membrane proteins that ultimately lead to a breakdown of the cellular structure of the bacteria [48]. Additionally, the interaction between the negatively charged substances of the microbial cell surface and the positively charged surface of the adsorbent exhibits cell wall lysis [49]. Hence, the composites have high antimicrobial action towards Gram-negative bacteria, compared to Gram-positive ones, as confirmed by the attained results.

### 3.6. Comparison with Other Materials

Comparison of fluoride and arsenite removal capacity of Fe-pPD and other iron-based sorbents is shown in Table 8. Significantly, different synthesis processes, compositions, and experimental processes of sorbents determine their efficiency towards pollutant ion removal. However, it was observed that iron-based sorbents have high removal potential towards concerned pollutants. From a social and economic point of view, the Fe-pPD composite seems to be a better option due to its multiple functionalities, efficiency, environmental, and experimental conditions in remediating these toxic metal ions as well as pathogens in contaminated water.

## 4. Conclusions

Fe-doped poly-phenylenediamine was successfully synthesized using chemical co-oxidative polymerization. Fe was successfully incorporated onto the pPD matrix as validated by different morphological characterizations. The synthesized Fe-pPD composite was evaluated for As^3+^ and F^−^ uptake in an aqueous solution. However, the study discovered the proposed adsorbent has the potential ability to remove As^3+^ and F^−^ effectively. The attained batch experiment data have shown that the assessed parameters such as contact time, adsorbent dose, pH, etc., have a significant effect on As^3+^ and F^−^ adsorption. The rate of adsorption of F^−^ and As^3+^ onto Fe-pPD composite best followed the pseudo-second-order kinetic model; thus, the uptake mechanisms of both pollutants by the Fe-pPD composite are due to the chemisorption process. However, in this study, the intra-particle plot obtained from the adsorption data shows that the adsorption phenomena occur in more than one step. This was the same for both F^−^ and As^3+^ adsorption processes results, indicating the uptakes of both pollutants were not controlled by only intra-particle diffusion. These phases show the systematic mechanisms of both the As^3+^ and F^−^ species in solution occurring through the boundary layer diffusion, intraparticle pore diffusion, as well as on the active sites across the external Fe-pPD composite surface. Comparatively, the adsorption of both As^3+^ and F^−^ by Fe-pPD displayed that the sorption process followed the Freundlich isotherm model with a higher affinity for F^−^ than As^3+^ based on the obtained n values, R^2^, Adj.R^2^, reduced chi-squares, and residual sum of squares values. Thus, the adsorption phenomenon occurred on a homogeneous layer, meaning the synthesized sorbent is multi-layer. In addition, thermodynamically, the removal process for both As^3+^ and F^−^ by the Fe-pPD was endothermic in nature with an increase in the degree of randomness as validated by the positive values of ∆H° and ∆S°, respectively. The synthesized Fe-pPD composite successfully portrayed effective antimicrobial action towards waterborne pathogens and economical viability as it can be reused just by mere washing with clean water.

## Figures and Tables

**Table 1:** BET analysis results of bare pPD and Fe-pPD.

BET Parameters	pPD	Fe-pPD
BET surface area (m^2^/g)	8.68	18.41
Pore volume (cm^3^/g)	0.02	0.07
Pore diameter (nm)	7.79	15.12
Average particle size (nm)	60.99	32.76

**Table 2:** CHNS results of Fe-pPD composite.

CHNS	N [%]	C [%]	H [%]	S [%]
Before application	14.9	52.96	4.315	2.54
After application	23.1	62.4	4.367	BDL

**Table 3:** Table for non-linearized kinetic parameters of F^−^ and As^3+^ onto the Fe-pPD composite.

Parameters	Pseudo-First Order		Pseudo-Second Order	
	F^−^	As^3+^	F^−^	As^3+^
*K*_1_	1.31	1.03	1.13	1.14
*Q_e_*	1.77	0.96	1.83	1.03
*R* ^2^	0.54	0.71	0.76	0.85
*χ* ^2^	0.03	0.21	0.01	0.15
*RMSE*	0.02	0.12	0.008	0.09

**Table 4:** Table for linearized kinetics models of F^−^ and As^3+^ onto the Fe-pPD composite.

Elovich			Intra-Particle Diffusion						
			Phase 1			Phase 2		Phase 3	
Parameters	F^−^	As^3+^	Parameters	F^−^	As^3+^	F^−^	As^3+^	F^−^	As^3+^
*β*	0.18	0.12	*C_i_*	0.42	1.53	0.78	1.07	0.98	0.8
*α*	4.08 × 10^3^	1.05 × 10^3^	*R* ^2^	0.53	0.69	0.52	1	0.94	0.87
*R* ^2^	0.96	0.86	*K*_1_	0.1	0.05	0.05	0.19	0.01	0.41

**Table 5:** Non-linearized adsorption isotherm values of F^−^ and As^3+^ onto the Fe-pPD composite.

Langmuir			Freundlich			
Temperature	Parameters	F^−^	As^3+^	Parameters	F^−^	As^3+^
298 K	*Q_m_*	6.79	1.86	*K_f_*	3.36	1.38
	*K_L_*	0.80	4.05	*n*	5.46	12.6
	*R* ^2^	0.83	0.82	*R* ^2^	0.87	0.65
	*Adj.R* ^2^	0.80	0.78	*Adj.R* ^2^	0.84	0.58
	*R_L_*	0.11	0.05	*χ* ^2^	0.89	0.05
	*χ* ^2^	1.15	0.03	*RSS*	4.46	0.24
	*RSS*	5.76	0.13			
303 K	*Q_m_*	5.55	1.67	*K_f_*	3.13	1.26
	*K_L_*	1.05	10.32	*n*	6.83	12.08
	*R* ^2^	0.85	0.60	*R* ^2^	0.85	0.67
	*Adj.R* ^2^	0.82	0.52	*Adj.R* ^2^	0.82	0.61
	*R_L_*	0.09	0.02	*χ* ^2^	0.57	0.04
	*χ* ^2^	0.56	0.05	*RSS*	2.85	0.22
	*RSS*	2.81	0.76			
313 K	*Q_m_*	4.02	1.72	*K_f_*	2.67	1.25
	*K_L_*	2.08	10.66	*n*	9.32	10.42
	*R* ^2^	0.72	0.39	*R* ^2^	0.82	0.51
	*Adj.R* ^2^	0.67	0.27	*Adj.R* ^2^	0.78	0.42
	*R_L_*	0.05	0.02	*χ* ^2^	0.28	0.12
	*χ* ^2^	0.42	0.15	*RSS*	1.38	0.61
	*RSS*	2.11	0.76			
323 K	*Q_m_*	4.04	2.17	*K_f_*	2.37	1.35
	*K_L_*	1.03	3.28	*n*	7.19	7.41
	*R* ^2^	0.43	0.44	*R* ^2^	0.56	0.54
	*Adj.R* ^2^	0.32	0.33	*Adj.R* ^2^	0.49	0.45
	*R_L_*	0.09	0.06	*χ* ^2^	1.18	0.26
	*χ* ^2^	1.54	0.32	*RSS*	5.91	1.31
	*RSS*	7.72	1.58			

**Table 6:** D-R isotherm model parameters of F^−^ and As^3+^ onto the Fe-pPD composite.

Temperature	D-R Model	F^−^	As^3+^
298 K	*R* ^2^	0.67	0.88
	*β*	9 × 10^−9^	4 × 10^−9^
	*E*	7.45	3.54
	*Q_max_*		1.82
303 K	*R* ^2^	0.72	0.68
	*β*	9 × 10^−9^	2 × 10^−9^
	*E*	7.45	5
	*Q_max_*	4.5	1.64
313 K	*R* ^2^	0.8	0.48
	*β*	6 × 10^−9^	1 × 10^−9^
	*E*	9.13	7.07
	*Q_max_*	3.6	1.66
323 K	*R* ^2^	0.58	0.58
	*β*	6 × 10^−9^	3 × 10^−9^
	*E*	9.13	4.08
	*Q_max_*	3.4	2.03

**Table 7:** Thermodynamic parameters of F^−^ and As^3+^ onto the Fe-pPD composite.

Parameters	F^−^	As^3+^
*∆H*° (kJ/mol^−1^)	1.3	0.57
∆*S°* (kJ/mol^−1^)	0.01	0.03
∆*G°* (kJ/mol^−1^)		
298 K	−9.19	−0.41
303 K	−9.35	−0.44
313 K	−9.68	−0.50
323 K	−10.01	−0.55

**Table 8:** Comparison F^−^ and As^3+^ removal capacity of Fe-pPD and other iron-based sorbents.

Pollutant	Sorbents	Concentration	Time	Dose	pH	Adsorption Capacity	Ref
As^3+^	Fe_3_O_4_-ʏ-Fe_2_O_3_	1.5 mg/L	100 min	0.4 g	2	3.69 mg/g	[47]
	Fe_3_O_4_	1 mg/L	24 h	5 g	2	46.06 mg/g	[50]
	αFe_2_O_3_	0.115 mg/L	50 min	-	-	95 mg/g	[51]
	Fe-pPD	5–100 mg/L	40 min	0.25	9	1.87 mg/g	This study
F^−^	Mg-Fe-La	10–150 mg/L	24 h	0.5 g	7	114.17 mg/g	[52]
	Fe(III)-Zr(IV)	2–50 mg/L	200 min	30 g	7	1.79 mg/g	[53]
	NasFeO	6 mg/L	90 min	0.5 g	7	1.20 mg/g	[54]
	Fe-pPD	5–100 mg/L	20 min	0.2 g	7	13.27 mg/g	This study
